# Phenotypic Transitions the Processes Involved in Regulation of Growth and Proangiogenic Properties of Stem Cells, Cancer Stem Cells and Circulating Tumor Cells

**DOI:** 10.1007/s12015-024-10691-w

**Published:** 2024-02-19

**Authors:** Magdalena Kulus, Maryam Farzaneh, Artur Bryja, Mojtaba Zehtabi, Shirin Azizidoost, Mahrokh Abouali Gale Dari, Afsaneh Golcar-Narenji, Hanna Ziemak, Mikołaj Chwarzyński, Hanna Piotrowska–Kempisty, Piotr Dzięgiel, Maciej Zabel, Paul Mozdziak, Dorota Bukowska, Bartosz Kempisty, Paweł Antosik

**Affiliations:** 1 Department of Veterinary Surgery, Institute of Veterinary Medicine, Nicolaus Copernicus University in Torun, Torun, Poland; 2 Fertility, Infertility and Perinatology Research Center, Ahvaz Jundishapur University of Medical Sciences, Ahvaz, Iran; 3 Division of Anatomy, Department of Human Morphology and Embryology, Wroclaw Medical University, Wroclaw, Poland; 4 Hematology and Oncology Research Center, Tabriz University of Medical Sciences, Tabriz, Iran; 5 Atherosclerosis Research Center, Ahvaz Jundishapur University of Medical Sciences, Ahvaz, Iran; 6 Department of Obstetrics and Gynecology, School of Medicine, Ahvaz Jundishapur University of Medical Sciences, Ahvaz, Iran; 7 Prestage Department of Poultry Science, North Carolina State University, Raleigh, NC USA; 8 Veterinary Clinic of the Nicolaus Copernicus University in Torun, Torun, Poland; 9 Department of Toxicology, Poznan University of Medical Sciences, Poznan, Poland; 10 Department of Basic and Preclinical Sciences, Institute of Veterinary Medicine, Nicolaus Copernicus University in Torun, Torun, Poland; 11 Division of Histology and Embryology, Department of Human Morphology and Embryology, Wroclaw Medical University, Wroclaw, Poland; 12 Department of Physiotherapy, Wroclaw University School of Physical Education, Wroclaw, Poland; 13 Division of Anatomy and Histology, University of Zielona Góra, Zielona Góra, Poland; 14 Physiology Graduate Faculty, North Carolina State University, Raleigh, NC USA; 15 Department of Diagnostics and Clinical Sciences, Institute of Veterinary Medicine, Nicolaus Copernicus University in Torun, Torun, Poland; 16 Department of Obstetrics and Gynecology, University Hospital and Masaryk University, Brno, Czech Republic

**Keywords:** Epithelial-mesenchymal Transition (EMT), Mesenchymal-epithelial Transition (MET), Circulating Tumor cell (CTC), Cancer stem Cells

## Abstract

Epithelial-mesenchymal transition (EMT) is a crucial process with significance in the metastasis of malignant tumors. It is through the acquisition of plasticity that cancer cells become more mobile and gain the ability to metastasize to other tissues. The mesenchymal-epithelial transition (MET) is the return to an epithelial state, which allows for the formation of secondary tumors. Both processes, EMT and MET, are regulated by different pathways and different mediators, which affects the sophistication of the overall tumorigenesis process. Not insignificant are also cancer stem cells and their participation in the angiogenesis, which occur very intensively within tumors. Difficulties in effectively treating cancer are primarily dependent on the potential of cancer cells to rapidly expand and occupy secondarily vital organs. Due to the ability of these cells to spread, the concept of the circulating tumor cell (CTC) has emerged. Interestingly, CTCs exhibit molecular diversity and stem-like and mesenchymal features, even when derived from primary tumor tissue from a single patient. While EMT is necessary for metastasis, MET is required for CTCs to establish a secondary site. A thorough understanding of the processes that govern the balance between EMT and MET in malignancy is crucial.

## Introduction

For many years, cancer has been recognized as a complex human disease characterized by both molecular and clinical aberrations [1]. Given the remarkable complexity and distinct characteristics of malignant tumors, a comprehensive and targeted investigation of its various aspects is crucial for a deeper understanding of the disease [2]. Metastasis is a critical hallmark of cancer that involves a complex interplay of molecular events, with tissue invasion being a crucial initial step in the process [3]. The spread of cancer cells in a collective manner highlights the importance of epithelial-to-mesenchymal transition (EMT), a process in which tightly-bound tumor cells acquire mesenchymal plasticity and they become more mobile [4]. This acquired capability enhances the ability of cancer cells to invade surrounding tissues and migrate to distant sites [2]. It is important to note that the process of EMT is not a clear-cut, binary transformation, but rather a spectrum where cells exhibit varying degrees of epithelial and mesenchymal features [5]. Notably, the cells that exist at the borderline between epithelial and mesenchymal states have been shown to circulate, colonize, and establish metastatic sites more efficiently [6]. Beyond its role in metastasis, EMT has also been implicated in conferring chemoresistance to cancer cells, highlighting the multifaceted nature of this process [7]. Conversely, mesenchymal-to-epithelial transition (MET) is a process where cancer cells revert to an epithelial state, and it is crucial for establishing successful metastasis by enabling dormant cells to proliferate and form secondary tumors [8]. However, it is important to note that MET is not simply the reverse of EMT, as the two pathways are regulated by distinct sets of mediators, leading to differences in their underlying molecular mechanisms [9, 10]. EMT and MET are fundamental biological processes that occur during tissue development and embryogenesis, and are essential for the formation of various organs and tissues [11]. In the context of tumor development, certain cancer cells acquire stem-like properties such as self-renewal capacity, resistance to apoptosis, and the ability to proliferate independently of external signals [12]. Cancer stem cells (CSCs) are known to be key players in the development of tumor heterogeneity and resistance to cancer therapy [13]. The correlation between EMT and cancer stemness has been established in several types of carcinomas, underlining the importance of understanding the crosstalk between these two processes in cancer biology [14]. Several transcription factors that play a role in the EMT process have also been shown to promote the acquisition of CSC properties, further highlighting the close relationship between these two processes in tumor development [15]. Furthermore, certain molecules such as IL-6 and TGF-β, which are secreted by various microenvironmental components, have been shown to enhance both EMT and stemness properties in cancer cells [16]. In order to establish a metastatic lesion, cancer cells must first intravasate into the bloodstream and travel through the circulatory system [17]. Circulating tumor cells (CTCs) have emerged as a valuable target for investigation due to their ease of accessibility and potential for predicting disease prognosis [18]. The detection of circulating tumor cells expressing mesenchymal phenotypes has been associated with several clinical parameters of the tumor, such as advanced disease stage and unfavorable prognosis [19]. The involvement of stem cells is indispensable for angiogenesis, as they can differentiate into different cell types within blood vessels or release exosomes that promote the growth of new blood vessels [20]. Given the critical role of CSCs in tumor angiogenesis, progression, and therapy resistance, targeting these mechanisms has emerged as a promising strategy for the development of novel cancer therapies [21, 22].

## Signaling Pathways Governing EMT and ETM during Stem Cells Growth, Proliferation, Differentiation, and Migration

Stem cells are a unique type of undifferentiated cells found in the human body that possess the remarkable ability to develop into multiple cell types, serving as the foundation for the growth, repair, and regeneration of diverse tissues, organs, and cells [23]. Stem cells are characterized by their capacity to differentiate into various cell types within an organism, as well as their ability to undergo unlimited self-renewal [24]. Stem cells are classified based on their differentiation potential, with the five major types including totipotent, pluripotent, multipotent, oligopotent, and unipotent stem cells, each with varying levels of developmental plasticity and therapeutic potential [25]. Stem cells can be classified based on their origin, with the most widely studied types including embryonic stem cells derived from the blastocyst, adult somatic stem cells generated during ontogenesis, fetal stem cells isolated from specific organs or tissues, tissue-specific embryonic stem cells, amniotic epithelial cells originating from the amniotic membrane of the human placenta, and umbilical cord epithelium derived from the epithelial amniotic membrane [26]. EMT as a vital cellular process has been well specified within embryonic evolution and it also is involved in different physiological events such as differentiative capacity of embryonic stem cell [27]. It has been reported that delocalization and reduction of E-cadherin, one of the principal initiation phenomenon of EMT, let to imperfect EMT where embryonic stem cells keep their undifferentiated condition meanwhile expressing different features of a mesenchymal-like phenotype [28]. EMT alteration is shown to be regulated through activation of main transcription factors of EMT such as SNAI1, SNAI2 whose roles are greatly modulated at transcription and translation. EMT initiation participates alterations in gene expression and activation of signals [29, 30]. A recent study revealed that low expression of E-cadherin elevated SNAI1 and SNAI2 expressions, induced low expression of several long non-coding RNAs (lncRNAs), such as MALAT1 and LINC-ROR, regulated the expression of tight and gap junction including occludin 1 and conexin 43, thereby increasing migration of human embryonic stem cells and β-catenin delocalization. Previous studies lead to a deeper understanding of the molecular events of an impermanent intermediate state and recognize numerous layers of molecular alterations that happened within partial EMT [28]. As mesenchymal stem cell has been known as a promising curative approach for acute lung injury, mechanistic consideration demonstrated that MSC inversed the EMT process via sequestering the activation of Hedgehog as well as NF-κB signal in injured lung cells, providing utility significance for acute lung injury therapy [31]. Previous studies reported that EMT can be induced via a modulatory network organized through a variety of pathways specifically transforming growth factor beta 1 (TGF-β1) as a crucial driver [32]. Currently, it has been found that exosomal mesenchymal stem cells inversed EMT through TGF-β1/Smad signaling and induced damaged endometrium repair [33]. Moreover, human umbilical cord mesenchymal stem cell was implicated in the attenuation of renal fibrosis through suppressing EMT and TGF-β/Smad signals [34]. Polycomb group (PcG) RING finger protein 5 (PCGF5) is shown to be implicated in epigenetic transcriptional suppression. It is informed that Pcgf5 deletion affected EMT during morphogenesis of embryonic stem cells and differentially impacted the gene expression of important developmental pathways such as Wnt, highlighting evidence that coordination of Pcgf5 with other fundamental developmental pathways arranged EMT as well as commitment of early cell lineage [35]. In addition, a growing body of evidences have informed that MET is connected to the properties of stem cells. It has been shown that MET along with EMT are necessary for immortalized oral epithelial cells to keep epithelial features of stem cells [36]. MET was also reported to be correlated with differentiative potential of hepatic stem cell in mice [37].

## Signaling Pathways Governing EMT and ETM during Cancer Stem Cells (CSCs) Growth, Proliferation, and Migration

Cancer stem cells (CSCs) were first recognized in hematologic malignancy and then isolated through expressing surface markers CD34 + and CD38-. They have an intense self-renewal capacity [38]. Their ability of expanding in a symmetrical splitting way leads to excessive cell growth induction that is eventually cause tumor formation [39]. Like normal stem cell, CSCs regulate signaling pathways which are participated in self-renewal process like Notch and Wnt/β-catenin pathways [40]. Along with their self-renewal capacity, CSCs can differentiate into various cell types. Once the modulatory balance between self-renewal and differentiation is eradicated, uncontrolled CSCs finally cause tumorigenesis. The colony-forming capacity of CSCs is also applied for identification and separation [41]. CSCs are also able to efficiently preserve malignant cells from apoptosis through activation of DNA repair potentials [42]. FOXC2 as a transcription factor that is increased in response to different EMT signals is a crucial determinant of mesenchymal and stem cell characteristics. Upregulation of FOXC2 was adequate to elevate CSC characteristics and spontaneous metastatic ability in transformed human mammary epithelial cells. Claudin-low/basal B breast malignant subtype that possess CSC along with EMT features has been shown to enrich in FOXC2. Therefore, FOXC2 or its correlated gene expression pattern may serve as an efficient target for anti-EMT-based treatments for the curative of claudin-low/basal B breast cancers or other EMT-/CSC-enriched malignancies [43]. Currently, two new notions have appeared in cancer biology: the function of CSCs in cancer initiation, and the collaboration of EMT in the metastatic diffusion of epithelial cancer cells. It was shown that cells expressing both stem and tumorigenic features of CSCs can be obtained from human mammary epithelial cells following Ras-MAPK activation. The acquisition of these stem and tumorigenic characters is driven by EMT induction. Various transcription factors and miRNAs have been involved in the molecular insights interlinking EMT to the acquisition of stem cell features, even though the accurate molecular streams remain largely undetermined. The latest research shows that the increase of EMT through ectopic expression of TWIST, TGFβ, or SNAIL therapy in immortalized and transformed human mammary epithelial cells leads to the acquisition of stem cell characteristics including self-renewal and tumor initiation [44, 45]. Additionally, chronic upregulation of the homeobox protein Six1 in the mouse mammary gland produced extremely aggressive cancers with an EMT phenotype, stem cell characteristics as well as activated Wnt pathway, informing crucial in vivo proof for the appearance of cells with merged EMT/CSC phenotypes [46]. Based on recent findings, diverse new EMT-enhancers that emerge to serve either upstream or together with the recognized EMT transcription factors have been participated in the transcriptional hierarchy of EMT and the confirmation of the emerged EMT/CSC phenotype [47]. Two current researches have confirmed the function of the niche in the induction of tumor development by affecting EMT-dependent advent of CSC features. First, the activating transcription factor 3 gene is an adaptive-response gene that may act to combine stromal signaling pathways from the tumor niche with the acquisition of emerged EMT/CSC characteristics in mammary epithelial cells [48]. Second, pathway via the urokinase-type plasminogen activator receptor can also stimulate EMT and elevate CSC features of exposed cancer cells to hypoxia [49]. The uncovering of miRNAs has subjoined an additional level of complication to the molecular networks modulating metastasis, EMT, as well as stemness [50, 51]. Compatible with the elevation of EMT as a vital early step in cancer metastasis, low expression of the miR-200 family has been comprehensively evidenced to occur within EMT and in aggressive breast malignancies. Moreover, ZEB1 has been reported to stimulate EMT through repressing members of miR-200 family at the aggressive front of pancreatic cancers, thereby resulting in the creation of migrating CSCs. Low expression of the miR-200 family have also been defined in normal animal and human stem cells and malignant CSCs, amplifying the molecular correlations between normal stem cells, CSCs along with the functions of the miR-200 family in modulating EMT and stemness [51]. EMT process has been shown to generate cells-enriched stem cell and CSC behaviors to introduce networks of signaling pathways that are more favorably used in the cellular products of an EMT. Pharmacologic suppression of protein kinase C α (PKCα) can selectively affect breast CSCs and EMT phenotype and that clinically efficient compounds repressing PKCα may confirm curatively helpful for curing special breast cancers [52]. Moreover, leptin signaling has been found to elevate cell viability, migratory and invading abilities, and CSC- and EMT-correlated gene expression in breast cancer. Thus leptin-correlated pathways affecting CSC and EMT may highlight novel targets as well as intervention approaches for reducing triple-negative breast cancer burden [53]. Arsenic predisposes human keratinocyte to neoplastic transformation. In this regard, as time course of neoplastic transformation passed within human keratinocyte cells, inhibitor nuclear factor-kappa B alpha (IκBα), IκB kinase β (IKKβ), nuclear factor κB (NF-κB) RelA activation as well as Snail level were induced whereas suppression of NF-κB RelA sequestered the arsenite-promoted EMT, CSC-like phenotype acquisition, and neoplastic transformation. Such findings inform that EMT and CSC-like phenotype acquisition that were regulated through IKKβ/IκBα/RelA pathway by Snail implicated in arsenite-fasilitated malignancies [54]. Fine particulate matter (PM2.5) has been tightly associated with morbidity and mortality induction of lung malignancy throughout the world. chronic exposure of alveolar and bronchial epithelial cells to PM2.5 led to the Notch signaling activation through enhancing of Notch1 and Hes1 expression, increased significant EMT event in terms of decreased E-cadherin expression and increased Vimentin expression along with evident CSC features in terms of increased ABCG2 and ALDH1A1 as cell-surface markers and SOX2 and OCT4 as self-renewal genes, highlighting the progressing process of cell malignant characteristics. Notch1 inhibition may cause negative regulation of EMT and CSC to repress invading and migratory potentials, thus putatively acting as a promising curative target for PM2.5 promoted lung malignancy [55]. Besides, bone morphogenetic protein (BMP) pathway has been reported to control CSC formation through affecting EMT. P123 as a new peptide agonist acting like BMP signaling can suppress transforming growth factor-β (TGF-β) and facilitated EMT in primary malignant cells. More importantly, P123 and BMP-7 inverse the EMT proceeding in human breast CSCs, and repress the ability of growth and self-renewal [56]. One aspect of debate is whether it is vital for CSCs to go through EMT in order to implant metastases. In vitro experiments showed that EMT-CSCs are much more aggressive compared to their epithelial counterpoints [57, 58]. For instance, PKD-1 pathway was implicated in the regulation of CSC-correlated gene signature expression and elevated self-renewal ability of CSC. PKD-1 signal is required for the CSCs subpopulation maintenance in pancreatic neuroendocrine malignancies at an intermediate state along the EMT process, thus resulting in a phenotype of CSC along with plasticity and incomplete EMT. So, inhibition of PKD-1 signaling may induce the deletion of subpopulations of CSC to restrict the development of pancreatic neuroendocrine malignancies, curative resistance and metastasis [59]. Six1 is a pivotal modulator of embryonic progression that needs interplay with the Eya family of proteins (Eya1-4) and their upregulation is noted in multiple tumors. Noticeably, both Six1 and Eya have been exerted to regulate breast cancer metastasis in an independent manner. It is reported that knockdown Eya2 deletion in breast carcinoma cells inversed Six1 ability to facilitate TGF-β pathway, as well as to promote properties correlated with EMT and CSCs, proposing that Six1-dependentm Eya2 process to regulate a variety of pro-metastatic features [60]. Twist is regarded as a main EMT transcriptional factor. CSC-like behaviors including tumorsphere formation along with ALDH1 and CD44 expression were shown to dramatically induce in Twist-upregulating cells which activated β-catenin and Akt pathways. So, β-catenin and Akt activation pathways are needed for the sustention of EMT-correlated stem cell-like behaviors [61].

## Circulating Tumor Cells—Recent Cellular and Clinical Advances with Regard to EMT and MET Processes

For decades, the capability of cancer cells to detach from the primary site, resist cell death and migrate by blood flow has established the concept of circulating tumor cell (CTC) [62, 63]. These cells bear outstanding features to keep viable in the bloodstream until they reach a convenient secondary location and grow as metastatic tumor. These properties and potential vulnerabilities have made CTCs an attractive landscape for cancer diagnosis, treatment and prognosis [62, 64]. However, a large proportion of CTCs perish in the circulation. Despite being derived from the primary tumor tissue, CTCs depict molecular diversities including stemness and mesenchymal features, even two distinct CTC from an individual patient may reveal different molecular characteristics [65]. In contrast to epithelial cells, mesenchymal cells do not need anchorage to neighboring counterparts and depict more plasticity and motility. Epithelial-to-mesenchymal transition of tumor cells includes acquisition of specific features to stay viable when detach from the primary tissue and circulate in the bloodstream. Some levels of EMT have been described in CTCs associated with stem-like features, disease progression and unfavorable treatment outcome [66]. Although EMT is necessary for invasion and migration of tumor cells, for an established metastatic cascade CTCs require to gain epithelial characteristics (MET) to extravasate and seed in the secondary site. Even E-cadherin loss was reported to be associated with reduced CTC survival and suppressed metastasis [67, 68]. Therefore, for a favored metastasis, there should be a balance between EMT and MET processes [65]. By evaluating N-cadherin, EpCAM, CD45, Snail and Vimentin on CTCs obtained from invasive breast carcinoma patients, Tashireva and colleagues found heterogeneous CTC phenotypes indicating EMT flexibility [69]. Another investigation of CTCs from breast cancer revealed that fluid shear stress promotes EMT in the epithelial CTCs and maintains mesenchymal CTCs by mechanistically activating JNK signaling. Induction of EMT downregulates PUMA enhancing CTCs’ survival in the bloodstream [70]. Besides the heterogeneity generated by phenotypic plasticity, the EMT that occurs across metastatic spread is molecularly complex and context-dependent and causes the diversity of CTCs which circulate at the same time. Many EMT-associated transcription factors (e.g., ZEB and Twist) and receptor tyrosine kinase (RTK) pathway (e.g., c-Met and Notch) molecules have been investigated so far [71]. In the setting of colorectal cancer, tumor cells presenting EMT characteristics modulate vascular barrier and increase permeability to form CTCs and induce metastasis. Dou et al. reported that exosomal miR-27b-3p derived from these cells targets VE-cadherin and p120 mRNAs disrupting the connection between endothelial cells. Interestingly, elevated blood exosomal miR-27b-3p levels were associated with higher CTC count and more invasive cancer phenotype among the patients [72]. Moreover, Wang et al. found that µ-opioid receptor agonists (MORAs) make metastasis of bladder cancer easier regarding EMT-CTC axis. They discovered that MORAs upregulate the EMT transcription factor Slug via activating PI3K/Akt pathway which facilitates CTC formation and metastasis. In parallel, patients receiving opioids before surgery had higher levels of CTCs [73]. Mesenchymal properties of CTCs could also predict the clinical features. A study conducted by Yin et al. revealed that rectal cancer patients with higher plasma EMT-CTCs had larger tumor size. However, there was no correlation between EMT-CTC amounts and TNM staging and vascular/perineal invasion [19]. Also, among HCC patients, CTCs presenting mesenchymal markers (EMT-CTC) had a significant impact on the patients’ prognosis that individuals bearing EMT-CTCs depicted shorter overall and relapse-free survival in several studies. A pooled study conducted by Orrapin et al. revealed higher recurrence rate in HCC patients with mesenchymal CTCs [74].

## A Review on Recent Findings of Epithelial-to-Mesenchymal Transition Signaling Principles

**Fig. 1:**
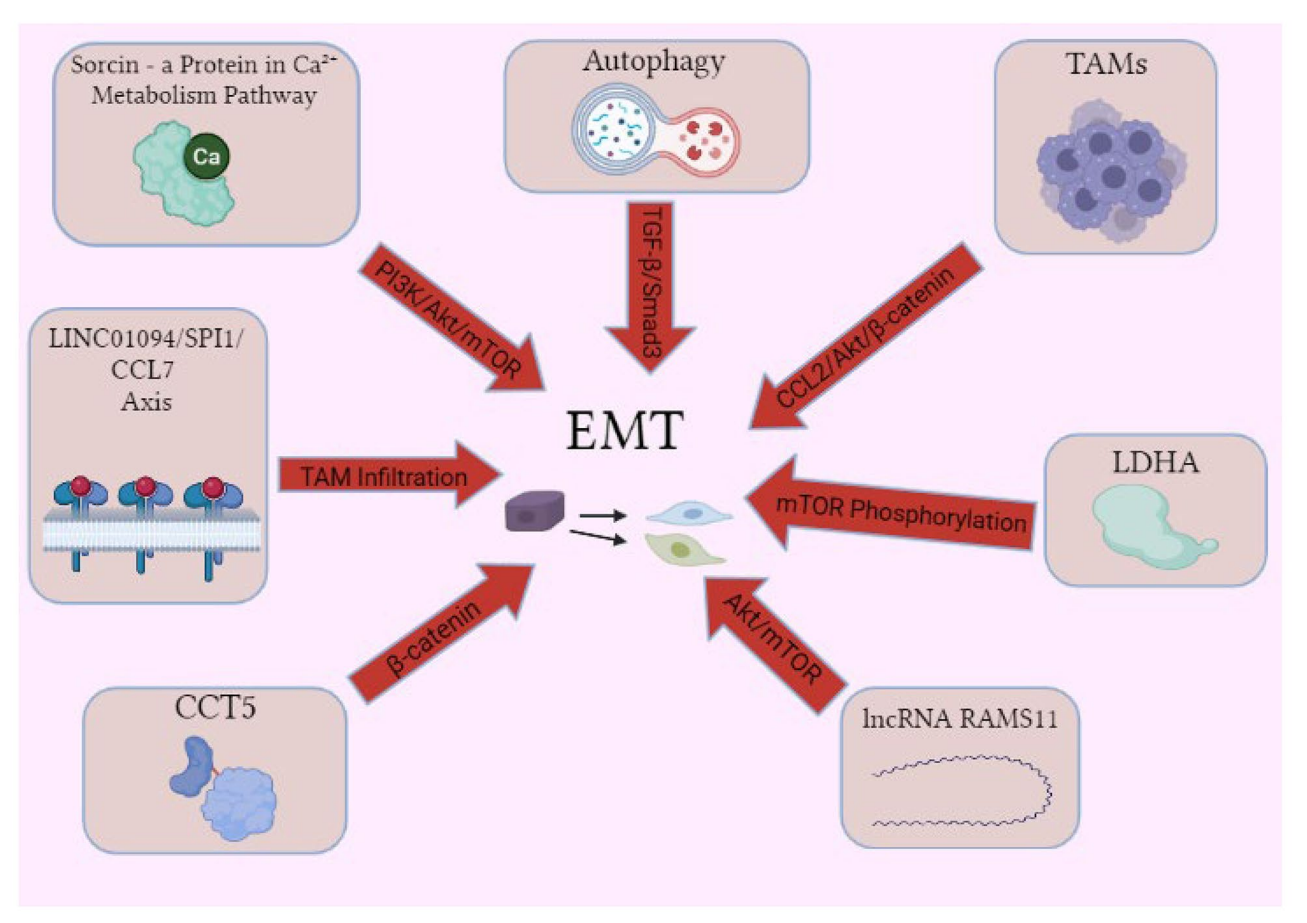
A concise overview of the molecular mediators that drive the promotion of epithelial-mesenchymal transition (EMT)

Malignant tissues exhibit astonishing diversity in phenotypic features of the constituent cells. The “heterogeneity” is derived from distinction between physiologic signaling networks. One of the major diversity contributors is acquirement of mesenchymal characteristics within carcinoma tissues. The EMT phenomenon have been studied for decades [75]. Conventionally, specific signaling routes (TGF-β, receptor tyrosine kinase (RTK), Notch, Wnt/β-catenin, Hedgehog, STAT3 and hypoxia) act to alter the expression profile of EMT-associated transcription factors (TFs) to induce and progress the EMT machinery. Some well-known EMT-associated TFs are Snail, ZEB and helix-loop-helix families as well as SOX4, PRRX1, YAP1/TAZ [76, 77]. As a model, Snail1 which is a member of Snail family and bear a fundamental role in EMT initiation is regulated by several TFs including NF-κB, LOXL2, HMGA2, PARP1 and Gli1. Snail1 itself modulates the expression of cell-to-cell interaction molecules such as E-cadherin, claudin, occludin, ZO-1, mucin1 and cytokeratin18. Matrix metalloproteinases are also regulated by Snail1 [77, 78]. Deregulation in calcium homeostasis is able to increase tumor invasion and migration by inducing EMT through multiple mechanisms such as extracellular matrix remodeling, invasion and integrin interplay. Sorcin (soluble resistance-related calcium-binding protein) is a calcium-binding protein with many physiologic roles. However, in cancer tissues, this protein is an oncogene associated with multi-drug resistance. Among the abundant processes, EMT induction is one of the suggested mechanisms established by PI3K/Akt/mTOR pathway [79, 80]. Macrophages residing and infiltrating tumor tissue (tumor-associated macrophages; TAMs) depict dual characters in cancer progression. The capability of killing tumor cells, mediating antibody-dependent tumor cytotoxicity and eliciting tumor necrosis in parallel to contribution in chemoresistance and cancer proliferation have made them promising therapy targets [81]. Recently, Chen et al. reported that there are high amounts of TAMs in human triple-negative breast cancer (TNBC) tissues. They also found that infiltrating M2 macrophages promote EMT in TNBC cells indicated by enhanced N-cadherin and vimentin expression as well as increased cell migration. The molecular pathway responsible for this phenomenon was β-catenin activation via C-C motif ligand 2 (CCL2)/Akt signaling. Also, patients with high TAM levels, demonstrated undesirable clinical features [82]. Another study conducted by Wu and colleagues revealed that in lung adenocarcinoma tissues, higher CCL7 expression was associated with increased TAM tissue infiltration and CCL7 knockdown resulted in suppressed migration and M2 polarization of macrophages. CCL7 transcription was promoted by LINC01094/SPI1 axis. Mesenchymal properties and mobility of lung adenocarcinoma cells were repressed following CCL7 knockdown representing TAMs as EMT inducers [83]. Autophagy, a catabolic machinery essential for preservation of cellular homeostasis, also is a double-edge sword in tumor progression and is known to be dysregulated in many carcinomas [84]. There are crosslinks between EMT and autophagy pathways. Aiming to investigate functions and mechanistic properties of lactate dehydrogenase A (LDHA) in papillary thyroid carcinoma (PTC) tissues, Hou et al. discovered that this enzyme is overexpressed in PTC tissues and is associated with invasive features and reduced patient recurrence-free and overall survival. They also revealed that LDHA contributes to cell proliferation, apoptosis inhibition and transcription of genes related to EMT. Moreover, LDHA inhibited autophagy. LDHA knockdown led to increased phosphorylated AMPK level and autophagy activation and EMT inhibition through ULK1 phosphorylation and mTOR dephosphorylation, respectively [85]. Previously, Bao et al. had found that paclitaxel-resembling agent alteronol can suppress cell proliferation/migration and induce autophagy via targeting and inhibition of Akt/mTOR pathway in melanoma cell lines. However, the enhanced autophagy may act as a cytoprotective property and promote EMT through TGF-β/Smad3 route indicating a need for simultaneous autophagy suppressor treatment [86]. LncRNAs and miRNAs play a remarkable role in positive and negative regulation of EMT process as well as crosstalk between EMT and autophagy providing a wide opportunity in cancer treatment landscape. MicroRNA-34a and miR-137 target and repress the expression of Snail in ovarian cancer cells leading to reduced EMT and sphere-forming capacity bringing more favorable clinical outcome. Also, miR-483-3p could reverse EMT to MET phenotype by targeting ITGB3 gene and thereby inhibiting downstream FAK/ERK pathways which causes reduction in lung cancer cell migration and metastasis [87]. LncRNA RAMS11 contributed to colorectal cancer (CRC) cell proliferation and migration in vitro. Also, downregulation of this lncRNA promoted autophagic and apoptotic activity of the CRC cells. However, RAMS11 downregulation was associated with reduced EMT indicator markers. The attributable molecular mechanism was reported to be Akt/mTOR signaling inhibition following AMPK pathway promotion [88, 89]. In addition, by studying EMT and autophagy markers (e.g., N-cadherin, vimentin, ATG5 and Beclin1) in gallbladder carcinoma cells, Li et al. depicted that miR-214/miR-3120 cluster suppressed EMT and autophagy and reduced tumor cell proliferation and invasion. E2F3 gene was demonstrated to be the target for this cluster [90]. Conventionally, Wnt/β-catenin pathway has been considered as an EMT driver. In a recent study performed by Li and colleagues, chaperon-containing TCP1 subunit 5 (CCT5) was shown to bind E-cadherin cytoplasmic domain overriding its interaction with β-catenin in gastric cancer cells. This results in β-catenin release into the nucleus and EMT enhancement. Therefore, CCT5 promotes gastric cancer proliferation and metastasis [91]. Also, TGF- β is a conventional activator of EMT which exerts through various pathways of which extracellular signal-regulated kinase (ERK) pathway is an example. ERK pathway plays role in the early steps of EMT. It has recently been reported that inhibition of ERK phosphorylation increases focal adhesion, cell contractility and alteration in expression of genes correlated to EMT induced by TGF- β1. Subcellular localization and phosphorylation of myocardin-related transcription factor-A (MRTF-A) is a suggested mediator which indicates a crosstalk between ERK and MRTF-A signaling in EMT promotion [92]. Figure 1 demonstrates the mentioned molecular mediators which lead to EMT promotion.

## Recent Findings on Proangiogenic Properties of CSCs—Molecular Mechanisms

**Fig. 2:**
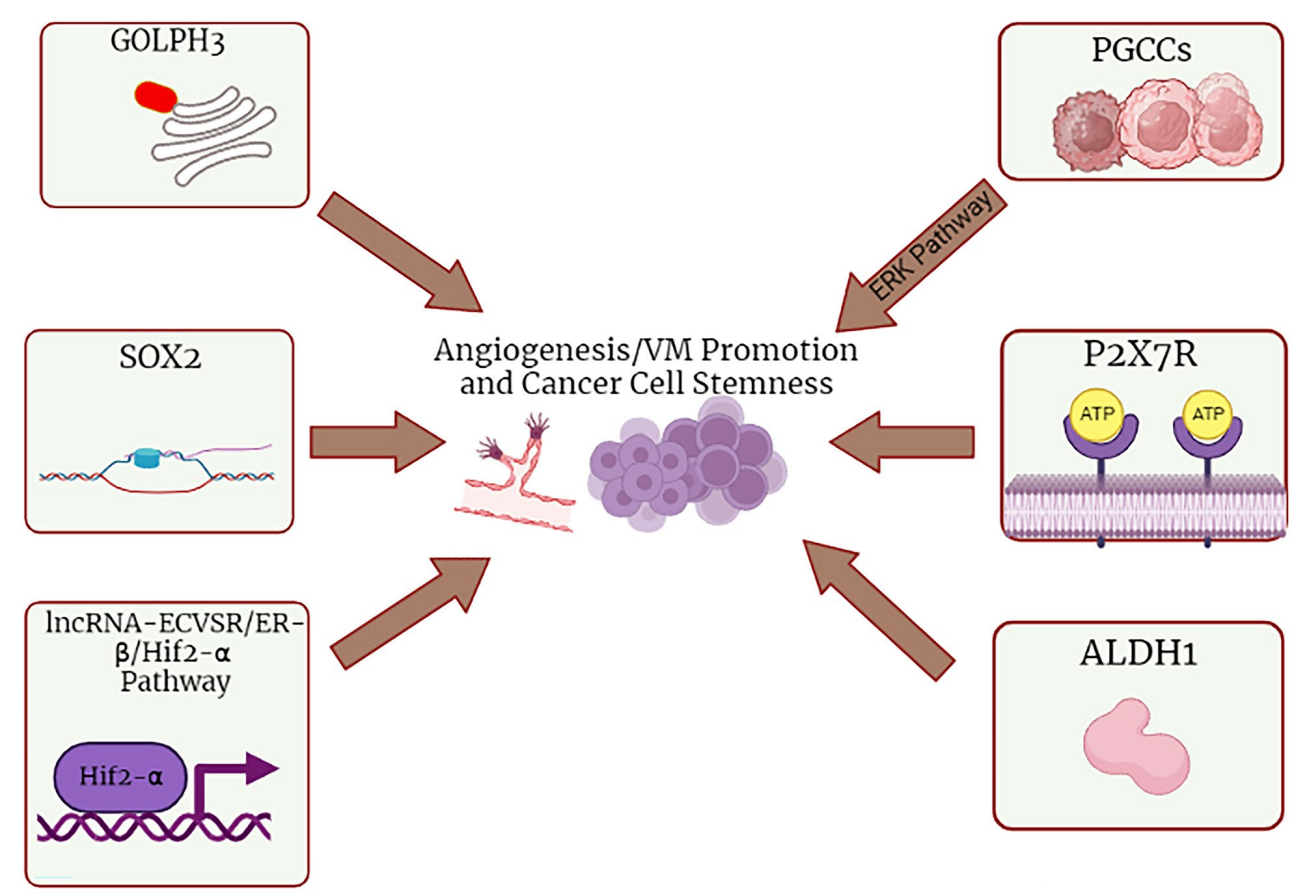
The molecular mechanisms involved in promoting angiogenesis/vasculogenic mimicry and cancer cell stemness

Formation of blood vessels or resembling structures is a well-studied emblem for solid tumors which keeps oxygen and nutrients accessible to the tumor cells. Tumors are able to develop and acquire metastatic capabilities in vascularized tissue. Regarding the presence of pro- and anti-angiogenic mediators in the regulation of angiogenesis, there should be an imbalance with pro-angiogenic dominancy to initiate new vessel formation [93]. However, some tumor types such as hepatocellular carcinoma and glioblastoma might hijack the existing blood vessels in alternative to new vessel formation which is called “vessel co-option” influencing disease fate by affecting therapy response and survival status [94]. Inhibiting new vessel formation and reducing tumor vascularity by targeting related pathway molecules (e.g., VEGF) has become a key step in cancer therapy regimens for years [93, 95]. In addition to neovascularization, vasculogenic mimicry (VM) is another mechanism recruited by high-grade tumors. This phenomenon is defined as origination of channels similar to blood vessels from non-angiogenic cancer cells. The VM could be established either in patterned matrix or tubular form. Unlike the tubular type, the patterned matrix VM does not contain a continual lumen [96]. The VM theory could, in part, explain cancer resistance to conventional cytotoxic or novel targeted therapies. The surviving malignant cells construct VM chambers to prepare oxygen and nutrients. Also, regarding the fact that hypoxia resulted from anti-angiogenic therapy may induce VM, tumors like glioblastoma may use this benefit to escape the angiogenesis-targeting treatments [97]. Many therapeutic approaches have been suggested to alter the VM process. For instance, histone deacetylase inhibitors (e.g., entinostat) were able to suppress formation of VM structures in a some cancer models [98]. By evaluating clinical samples from glioblastoma patients, Jia et al. revealed that expression level of oncogene Golgi phosphoprotein 3 (GOLPH3) was positively correlated to VM and a higher pathological grade. Furthermore, after GOLPH3 knock down in glioblastoma cell line, they observed that these cells expressed higher E-cadherin and lower MMP2. These results suggest interconnection between EMT and VM phenomena and GOLPH3 as a therapy target [99]. Cancer cell stemness plays a crucial role in both neovascularization and VM. Melanoma cells forming vessel-like structures display pluripotent embryonic genotype. Also, HCC cells lining the VM tubes were found to demonstrate stemness factors SOX2 and OCT4 [100]. Also, elevated level of CSC marker CD44 is associated with expression of vascular adhesion protein VE-cadherin and VM presence in oral squamous cell carcinoma tissues [101]. Polyploid giant cancer cells (PGCCs) are a subgroup of dormant tumor tissue cells which exhibit CSC features and facilitate tumor recurrence. Cheng et al. found that EBV can promote VM formation in nasopharyngeal carcinoma by enhancing stemness in PGCCs. Mediated by LMP2A, activation of ERK pathway was reported to be responsible. Promotion of EMT was also vital for VM formation [102]. Sunitinib, a tyrosine kinase inhibitor approved for treatment of renal cell carcinoma (RCC), acts mainly through inhibition of angiogenesis, but resistance occur in most cases. Recently, He et al. demonstrated that sunitinib might represent adverse effect of VM promotion in RCC. This effect happens through lncRNA-ECVSR/estrogen receptor β (ER-β)/Hif2-α signaling pathway that sunitinib increases expression of lncRNA-ECVSR. The mentioned pathway ultimately enhances CSC phenotype and VM formation. These findings suggest the necessity of alternative and combined treatment approaches which targets VM in the RCC therapy regimens [103]. ALDH1 is a well-known marker of CSCs among solid cancers. By evaluating TNBC cell line, Izawa et al. found that ALDH + cells had higher capacity to form VM structures compared to ALDH- cells [104]. Catulin is an α-catenin homologue that its overexpression is associated with increased migratory potential and EMT. In another research on TNBC cell line, Gielata et al. revealed that high catulin levels correlate with enhanced tumor progression and invasion. By developing a catulin reporter system, they discovered that catulin and the mesenchymal marker vimentin are positively correlated in expression. Moreover, after injecting cells expressing catulin reporter into immunocompromised mice, the catulin reporter system marked invading cancer cells at the tumor border and the cells around newly generated vasculature as well as the small population of MCAM-positive cells participating in VM. This study introduces catulin as a potential linker between pathways leading to breast cancer cell invasiveness which interacts with different CSC and cell adherent molecules [105]. All the aforementioned data indicate remarkably complex interlink between the CSC theory, VM machinery and EMT process in solid cancers which still remains a wide field of investigation and attractive therapeutic target. It is well documented that tumor angiogenesis is promoted by CSCs through several mechanisms. Expression and secretion of pro-angiogenic factors like VEGF and angiopoietin, recruiting fibroblasts and macrophages to secrete pro-angiogenic mediators and differentiation into vascular progenitor and endothelial cells are prominent examples. In a recently conducted study by Zhu et al. gastric CSCs were reported to possess higher motility and invasion capability as well as increased vascular loop formation potential than gastric cancer cells without stemness markers [106]. SOX2 functions as a stem cell TF that maintains CSC properties. Expression of SOX2 is associated with higher aggressiveness and poor outcome among CRC patients. Chen and colleagues reported that enhanced SOX2 expression is correlated with higher VM and angiogenesis in CRC cells and knockdown of SOX2 hindered CSC features, angiogenesis and VM in both in vivo and in vitro analyses. Also, SOX2 levels were positively correlated with endothelial markers CD31 and VE-cadherin. Ultimately, this study revealed that miR-450a-5p is an upstream regulator for SOX2 which in turn modulates CRC stemness and angiogenesis [107]. Eventually, in a combined in vitro and mouse orthotopic model investigation by Yang et al., P2X purinoceptor 7 (P2 × 7R) was found to promote CRC cell migration and proliferation by recruiting TAMs and activating cytokine upregulation via NF-κB pathway. Also, P2 × 7R amplified ALDH1 level and CSC properties. Concurrent angiogenesis as well as CD31 and VEGF expression were promoted due to P2 × 7R amplification, as well [108]. Figure 2 outlines the aforementioned molecular mechanisms participating in angiogenesis/VM and cancer cell stemness promotion.

## Data Availability

Not applicable.
